# Public Attitudes Regarding Trade-offs Between the Functional Aspects of a Contact-Confirming App for COVID-19 Infection Control and the Benefits to Individuals and Public Health: Cross-sectional Survey

**DOI:** 10.2196/37720

**Published:** 2022-07-20

**Authors:** Seiji Bito, Yachie Hayashi, Takanori Fujita, Shigeto Yonemura

**Affiliations:** 1 Division of Clinical Epidemiology National Hospital Organization Tokyo Medical Center Tokyo Japan; 2 Department of Health Policy Management Keio University School of Medicine Tokyo Japan; 3 The Graduate Schools for Law and Politics University of Tokyo Tokyo Japan

**Keywords:** internet questionnaire survey, contact-confirming app, COVID-19, privacy, ethics in public health, health application, application development, health service, mobile phone, survey platform, public health, digital information, privacy, health information

## Abstract

**Background:**

It is expected that personal health information collected through mobile information terminals will be used to develop health strategies that benefit the public. Against this background, several countries have actively attempted to use mobile phones to control infectious diseases. These collected data, such as activity logs and contact history, are countermeasures against diseases such as COVID-19. In Japan, the Ministry of Health, Labor, and Welfare has developed and disseminated a contact-confirming app (COVID-19 Contact-Confirming Application [COCOA]) to the public, which detects and notifies individuals whether they have been near someone who had subsequently tested positive for COVID-19. However, there are concerns about leakage and misuse of the personal information collected by such information terminals.

**Objective:**

This study aimed to investigate the possible trade-off between effectiveness in preventing infectious diseases and infringement of personal privacy in COCOA. In addition, we analyzed whether resistance to COCOA would reduce if the app contributed to public health or if a discount was provided on mobile phone charges.

**Methods:**

A cross-sectional, quantitative survey of Japanese citizens was conducted using *Survey Monkey*, a general-purpose web-based survey platform. When developing the questions for the questionnaire, we included the installation status of COCOA and recorded the anxiety stemming from the potential leakage or misuse of personal information collected for COVID-19 infection control. The respondents were asked to rate various factors to determine their perceptions on a 5-point scale.

**Results:**

In total, 1058 participants were included in the final analysis. In response to the question of whether the spread of the disease was being controlled by the infection control measures taken by the government, 25.71% (272/1058) of the respondents answered that they strongly agreed or agreed. One-quarter of the respondents indicated that they had already installed COCOA. This study found that the sense of resistance to government intervention was not alleviated by the benefits provided to individuals when using the app. The only factors that were positively associated with the response *absolutely opposed to use* of the app, even with a discount on mobile phone use charges, were those regarding leaks and misuse of personal information, which was true for all functions (function A: odds ratio [OR] 1.8, 95% CI 1.3-2.4; function B: OR 1.9, 95% CI 1.5-2.6; function C: OR 1.8, 95% CI 1.4-2.4).

**Conclusions:**

Public organizations need to emphasize the general benefits of allowing them to manage personal information and assure users that this information is being managed safely rather than offering incentives to individuals to provide such personal information. When collecting and using citizens’ health information, it is essential that governments and other entities focus on contributing to the public good and ensuring safety rather than returning benefits to individual citizens.

## Introduction

### Background

With the development of information technology, the use of big data has become more common. Indeed, there are many examples where big data have been successfully used to promote general public health by referring to the personal information collected via the data terminals of smartphones, as smartphones are carried around by people everywhere and form a part of their lives [1,2].

Against this background, several countries have actively attempted to use mobile phones for infectious disease control by launching, as well as promoting the download of, apps to collect data, such as activity logs and contact history, as a countermeasure against COVID-19. Each country has unique methods for doing so [3,4]. For example, in South Korea and Singapore, the government has taken the lead in developing and disseminating contact-tracing apps that track the history of individuals who are infected and established the necessary system to rapidly share data with municipalities and medical institutions [5-7].

Although the situation in early 2022—in which unlimited personal information could be uploaded to a specific information cloud or similar means for secondary use—might have been convenient, it involved various ethical, legal, and social problems [8-10]. For example, many contact-tracing apps developed to prevent the spread of COVID-19 have GPS-tracking capabilities, and the information collected by these apps includes detailed movement records of individuals. If this information were to be leaked or used for purposes other than as intended, an individual’s privacy would be severely compromised. Moreover, because of the nature of the information, the purpose for which it is to be used must be communicated, along with details about the agencies managing the information and the scope of information use [11]. Wacksman [12] warned that the unprecedented demands stemming from the response to the spread of COVID-19 pose new risks, such as the collection of vast amounts of data and the risk of increasing demands for data sharing that go beyond the intended purpose.

In Japan, the Ministry of Health, Labor, and Welfare has developed and disseminated a contact-confirming app (COVID-19 Contact-Confirming Application [COCOA]) to the public, which notifies individuals whether they have been near an individual who has tested positive for COVID-19. The Ministry of Health, Labor, and Welfare explains the purpose of COCOA on its website as follows:

With knowledge of the possibility that an individual was in contact with a “positive” individual, users can receive early support from public health centers, such as by undergoing a test. As the number of users increases, it is hoped that this initiative will help prevent the spread of infection.

As of April 2022, there have been >32.5 million downloads of COCOA, and it is thought that approximately 1 in 5 people living in Japan have installed the app on their mobile device or devices. In Japan, COCOA is the most widely used contact verification app, and citizens have only 2 choices: installing or not installing COCOA.

COCOA was developed as a Bluetooth-based exposure notification app rather than a contact-tracing app. Thus, current COCOA functions do not have the ability to track moving individuals through GPS or collect personal movement logs. If users who have installed this app on their mobile device come into close contact (within 1 m for ≥15 minutes) with an individual who is COVID-19 positive (positive registration is confirmed by self-report), the Bluetooth communication function detects this and informs the person that they have been in high-risk contact with an individual who is COVID-19 positive.

The information delivered through the notification does not include the location information of the app user who is infected—only a contact code is exchanged when a user contacts another app user’s smartphone. Owing to the nature of COCOA’s functions, there is very little risk of personal information being shared with government authorities, and the possibility of private information being externally leaked or used for other purposes is also considered to be extremely low [13].

However, whether COCOA functions effectively as an infection control measure against COVID-19 warrants further investigation, as there appear to be 3 reasons for its likely dysfunction. First, the app involves a double opt-in by users in terms of positive result registration through COCOA, which requires both the installation of COCOA on mobile devices and the proactive registration of a positive result (as sharing test results is not mandatory). Consequently, it is assumed that there are many undetected cases for which no alerts are issued, even when a person has actually been in contact with an individual who is COVID-19 positive [13,14]. Second, the app cannot collect data on people’s movements, which makes it almost impossible for authorities to intervene directly or implement specific strategies, such as infection control, using the log data. Third, even for an alert indicating such contact, there is no function to suggest specific actions to be taken afterward; thus, the app cannot facilitate effective behavioral changes at the individual level to prevent further infections [13,14]. Several empirical investigations into the attitudes of actual users (citizens) of contact-tracing apps and exposure notifications toward the future use of such apps (regarding the balance between risks related to the leakage of private information, the use of such apps for other purposes, and the benefits to public health and individuals) have been conducted since the start of the COVID-19 pandemic [15-19]. In a survey aimed at examining why some Australian citizens had not downloaded the app launched by their government, Thomas et al [18] reported that citizens were concerned about the app’s technical functionalities and privacy and were distrusting of the government. According to a citizen survey on COCOA conducted by Machida et al [15], the main concerns that inhibited app use were identified as *lack of knowledge on how to use it*, *privacy concerns*, *doubts about the effectiveness of the app*, and *concerns about battery consumption and communication costs*. By contrast, there is a lack of international evidence on citizens’ acceptance of apps when there is a trade-off between the app’s features and the associated personal and public health benefits.

### Objectives

In this study, we conducted a quantitative survey of Japanese citizens to identify the extent to which they were resistant to features that could be implemented in contact-confirming apps. This study investigates the possible trade-off between the effective prevention of infectious diseases and infringements of personal privacy. In addition, we analyzed whether this resistance would decrease if users perceived a contribution in terms of public health or if they received a discount on their mobile phone charges in exchange for app use.

## Methods

### Study Design and Participants

In this study, we conducted a cross-sectional web-based survey using *Survey Monkey*, a general-purpose web-based survey platform [20]. The target population was set as the general population, aged ≥18 years, living in Japan, and having their own mobile devices. We recruited participants with a target of 1000 respondents. For this, we invited a sample of residents who fit the general target population requirements from among the survey monitors of the contracted research company, Asmarq.co.jp [21]. A sample of 1300 respondents was initially recruited, and if the final number of respondents did not reach 1000, additional samples were added. This process ended when the number of respondents exceeded 1000. The survey was anonymous as the researcher could track neither the respondents nor the nonrespondents. The researcher did not pay any incentives directly to the respondents; however, a sampling fee was paid to the research company. Participants were required to check the details and overview of the survey presented on the website. The actual survey commenced once they provided informed consent. The survey was conducted in June 2021.

### Instruments

A set of multiple-choice questions was developed to accomplish the objectives of this study through a cross-sectional survey on the internet. The specific survey items are listed in Multimedia Appendix 1.

As independent variables were assumed to be associated with dependent variables, we asked about the respondents' characteristics, frequency of use of social networking sites (SNSs), COVID-19 infection history, fears related to COVID-19 infection, level of adherence to infection prevention behaviors (behavioral restrictions and mask wearing), and their assessment of government agencies and hospital responsibility for infectious disease prevention.

As dependent variables, we tried to include the installation status of COCOA, as well as record respondents’ anxiety regarding potential leakage or misuse of personal information collected for COVID-19 infection control. Respondents were asked about their anxiety on a 5-point scale from *strongly agree* to *strongly disagree*, with *neutral* as their neutral option. In addition, respondents were asked about their degree of resistance to the use of the app if the following 3 functions (that COCOA does not currently have) were added:

- Function A: If a respondent is found to have COVID-19, information on the respondent’s infection would be provided to the authorities via a medical institution and reflected in COCOA notifications.
- Function B: In addition to function A, this function would use the location information function of smartphones to track and prompt individuals to take specific actions, such as requesting a medical examination.
- Function C: All movement data of people who have installed the app would be recorded through their mobile devices and aggregated by the government; these data would be used by the government to plan and implement specific infection prevention measures.

The respondents were asked to indicate their level of resistance to the use of the app if the various functions were added on a 5-point scale: *absolutely opposed to use*, *significant resistance to use*, *some resistance to use*, *not much resistance to use*, and *no resistance to use*. Finally, we asked about the resistance to each function when it was understood that the addition of functions A, B, or C would bring about a trade-off in benefits to individuals versus the public. Specifically, participants were asked about their resistance to each feature in the following cases:

- Condition 1: if 50% of the population uses the app with each feature added, the degree of spread of the infection would be halved compared with nonuse of the app.
- Condition 2: if installing the app with each feature resulted in a discount on their monthly mobile phone bill; the options were set as approximately ¥200 (US $1.48) per month, ¥500 (US $3.70) per month, ¥1000 (US $7.29) per month, ¥2000 (US $14.78) per month, ¥3000 (US $22.17) per month, ¥5000 (US $36.95) per month, and would not want to use it irrespective of the discount.

### Data Collection

Participants sampled by the survey company visited Survey Monkey’s website to take the survey we created. Thereafter, respondents were informed via the web about the purpose of the survey and the advantages and disadvantages of participating; only those who agreed to participate became respondents. The survey ended when the number of respondents exceeded 1000. Responses to the web-based survey were collected in the cloud, and the individual forms were downloaded by the principal investigator.

### Statistical Analysis

SPSS (version 27; IBM Corp) was used for statistical analysis. All data entered for the survey, including age, were categorical. Descriptive statistics included descriptions of all independent and dependent variables, including patient characteristics. Logistic regression analysis was performed to analyze the factors that could explain the dependent variables. In performing the logistic regression analysis, we attempted to dichotomize the dependent variables into *0* and *1*. As a criterion for dichotomization, we categorized anxiety regarding leakage or misuse of personal information collected for COVID-19 countermeasures between *agree* and *neither*. In addition, resistance to the proposed newly added functions was dichotomized between *absolutely opposed to use* and *significantly resistant to use*. The former option was dichotomized to determine whether a person had a small amount of anxiety. For the latter, we considered the presence of a clear and strong refusal to use the service as an important threshold for attitude. All statistical tests were 2-tailed, and *P*≤.05 was set as the threshold for statistical significance.

### Ethics Approval

This study was reviewed and approved by the Biomedical Research Ethics Committee of the National Hospital Organization Tokyo Medical Center in May 2021 (approval number R21-030).

## Results

### Participants’ Sociodemographic Information

We recruited 1300 people through a research company, of whom 1191 (91.62%) accessed the survey website. The eligibility criteria were being aged ≥18 years, living in Japan, and having their own mobile phones. Of the 1191 individuals, 1096 (92.02%) met the eligibility criteria, and after excluding those who did not give consent or dropped out, 1058 (88.83%) participants were included in the analysis.

The basic characteristics and frequency of SNS use by the respondents are presented in Table 1. Regarding respondents’ age, 8.82% (93/1058) were aged ≤34 years, 71.73% (759/1058) were aged 35 to 64 years, and 19.48% (206/1058) were aged ≥65 years. Regarding gender, 43.37% (459/1058) were female; regarding marital status, 66.6% (705/1058) were married; and regarding the number of people living together, 16.41% (174/1058) lived alone. The proportion of respondents who had personally contracted COVID-19 was 1.12% (12/1058), whereas the proportion of respondents whose family or close friends had been infected was 3.2% (34/1058). The frequency of SNS use (ie, Twitter, Facebook, and Instagram) was as follows (in the order *not used*, *used several times per month or less*, and *used several times per week or more*): 46.34% (487/1058), 20.6% (218/1058), 33.41% (353/1058) for Twitter; 52.32% (553/1058), 26.59% (281/1058), 21.1% (223/1058) for Facebook; and 58.78% (622/1058), 16.91% (179/1058), and 24.3% (257/1058) for Instagram.

**Table 1:** Respondent demographics (N=1058).

Characteristics and response options				Number of responses, n (%)
**Basic characteristics**				
	**Age (years)**			
		≤34	93 (8.8)	
		35-64	759 (71.7)	
		≥65	206 (19.5)	
	**Gender**			
		Female	459 (43.4)	
		Male	599 (56.6)	
	**Marital status**			
		Married	705 (66.6)	
		Unmarried	353 (33.4)	
	**Households**			
		Living alone	174 (16.4)	
		2 people	365 (34.5)	
		≥3people	519 (49.1)	
**COVID-19 infections**				
	**Respondent**			
		No	1028 (98.9)	
		Yes	11 (1.1)	
	**Family and close friends**			
		No	995 (96.8)	
		Yes	33 (3.2)	
**Use of SNS^a^ (frequency)**				
	**Twitter**			
		Does not use	486 (46)	
		Several times per month or less	218 (20.6)	
		Several times per week or more	353 (33.4)	
	**Facebook**			
		Does not use	552 (52.3)	
		Several times per month or less	281 (26.6)	
		Several times per week or more	223 (21.1)	
	**Instagram**			
		Does not use	620 (58.8)	
		Several times per month or less	178 (16.9)	
		Several times per week or more	256 (24.3)	
**Degree of compliance with preventative behaviors**				
	**Wearing masks when out**			
		I strictly adhere to this	716 (69.1)	
		I adhere to this with some exceptions	250 (24.1)	
		I am neutral to this	51 (4.9)	
		I do not really adhere to this	9 (0.9)	
		I do not ever adhere to this	10 (1)	
	**Restrictions on activities such as meetings**			
		I strictly adhere to this	598 (56.9)	
		I adhere to this with some exceptions	333 (31.7)	
		I am neutral to this	85 (8.1)	
		I do not really adhere to this	17 (1.6)	
		I do not ever adhere to this	18 (1.7)	
**Assessment of authorities in controlling the spread of infections**				
	**National and prefectural governments**			
		Strongly agree	55 (5.2)	
		Agree	217 (20.6)	
		Neutral	356 (33.8)	
		Disagree	237 (22.5)	
		Strongly disagree	188 (17.9)	
	**Local health centers**			
		Strongly agree	57 (5.4)	
		Agree	267 (25.5)	
		Neutral	464 (44.4)	
		Disagree	162 (15.5)	
		Strongly disagree	96 (9.2)	
**Assessment of authorities in controlling the spread of infections**				
	**Medical institutions such as hospitals**			
		Strongly agree	79 (7.5)	
		Agree	345 (32.8)	
		Neutral	456 (43.3)	
		Disagree	103 (9.8)	
		Strongly disagree	69 (6.6)	
**Assessment of measures against infection**				
	**National and prefectural governments**			
		Strongly agree	61 (5.8)	
		Agree	269 (25.6)	
		Neutral	337 (32.1)	
		Disagree	192 (18.3)	
		Strongly disagree	191 (18.2)	
	**Local health centers**			
		Strongly agree	90 (8.6)	
		Agree	372 (35.7)	
		Neutral	387 (37.2)	
		Disagree	121 (11.6)	
		Strongly disagree	72 (6.9)	
	**Medical institutions like hospitals**			
		Strongly agree	128 (12.2)	
		Agree	413 (39.5)	
		Neutral	374 (35.8)	
		Disagree	75 (7.1)	
		Strongly disagree	56 (5.4)	
**Fear of respondent becoming infected**				
	**In terms of health deterioration to oneself**			
		Strongly agree	378 (35.8)	
		Agree	437 (41.5)	
		Neutral	144 (13.6)	
		Disagree	66 (13.6)	
		Strongly disagree	30 (6.3)	
	**In terms of transmission to one’s family or friends**			
		Strongly agree	422 (2.8)	
		Agree	414 (40.1)	
		Neutral	147 (39.3)	
		Disagree	39 (14)	
		Strongly disagree	31 (2.9)	
	**In terms of transmission to other people**			
		Strongly agree	367 (34.8)	
		Agree	429 (40.7)	
		Neutral	176 (16.7)	
		Disagree	52 (4.9)	
		Strongly disagree	31 (2.9)	
**Anxiety regarding the handling of one's personal information**				
	**In terms of data leaks and misuse**			
		Strongly agree	135 (12.9)	
		Agree	296 (28.3)	
		Neutral	386 (36.9)	
		Disagree	178 (17)	
		Strongly disagree	51 (4.9)	
**Use of COCOA^b^**				
	**Have you installed COCOA?**			
		Yes	259 (24.9)	
		No	782 (75.1)	

^a^SNS: social networking site.

^b^COCOA: COVID-19 Contact-Confirming Application.

### Awareness of COVID-19 and Infectious Disease Control

The respondents’ attitudes toward COVID-19 and infection control measures are shown in Table 1. Respondents were asked how strictly they adhered to the government’s requests to wear a mask when going out and restrict their activities, such as attending meetings in person. Approximately 93.21% (986/1058) and 88.59% (937/1058) of respondents said that they strictly adhered to or adhered to the requests with some exceptions for wearing masks and for restricting activities such as meetings, respectively.

In response to the question of whether the spread of the disease was being controlled by the infection control measures taken by the government and various prefectures, 25.8% (273/1058) of respondents answered that they *strongly agreed* or *agreed*. When asked if the government and prefectures were conducting infection control measures with a strong sense of responsibility, 31.41% (332/1058) of respondents answered that they strongly agreed or agreed. When asked how fearful they were of harm to their own health, the transmission of the disease to their family and friends, or transmission to others if they were to contract COVID-19, the respondents answered that they either *strongly agree*” or *agree* with these 3 scenarios at 77.28% (818/1058), 79.43% (840/1058), and 75.5% (799/1058), respectively. When asked whether they were concerned that personal information and other data collected as part of the countermeasures against COVID-19 by the national and prefectural governments would be leaked or misused for other purposes, 41.17% (436/1058) of respondents answered that they *strongly agree* or *agree*.

One-quarter of the respondents indicated that they had already installed COCOA. They were given an overview of the functions of the current COCOA. Once each of the 3 aforementioned functions (A, B, and C) were added, the percentage of respondents who said that they would be absolutely opposed to its use or had significant resistance to its use was 27.31% (289/1058) for function A and 31.4% (332/1058) for function B. The difference compared with when only function A was added was not significant. With the addition of function C, this percentage was 33.63% (355/1058).

### Respondents’ Resistance to the Additional Functions, Even With Benefits and Trade-offs

The percentage of respondents who said they were *absolutely opposed to use* of the app even if half of the population used it with functions A, B, and C (enabling the degree of spread to be reduced to half of what it would be if the app were not used) was 11.6% (123/1058) for function A, 13.33% (141/1058) for function B, and 13.71% (145/1058) for function C. The percentage of respondents who said that they were *absolutely opposed to use* of the app, even if the effect of using it was to halve the spread of infections, was 10.36% (110/1058) for function A, 12.21% (129/1058) for function B, and 12.43% (131/1058) for function C. In response to the question “Would you accept the use of the app with each of the functions added, if the following reductions in mobile phone usage charges were applied?” the respondents who selected “I would not want to use the app irrespective of the discount” was 34.62% (366/1058) for function A, 36.77% (389/1058) for function B, and 37.3% (395/1058) for function C (Table 2).

**Table 2:** Resistance to using the COVID-19 Contact-Confirming Application under various conditions when functions are added to the current version (N=1058).

Condition and response option			Function A (responses), n (%)		Function B (responses), n (%)		Function C (responses), n (%)
**Feature addition only**							
	No resistance to use	141 (13.5)		123 (11.9)		124 (12)	
	Not much resistance to use	282 (27.1)		251 (24.2)		243 (23.4)	
	Some resistance to use	334 (32.1)		337 (32.5)		321 (31)	
	Significant resistance to use	164 (15.7)		187 (18.1)		206 (19.9)	
	Absolutely opposed to use	121 (11.6)		138 (13.3)		142 (13.7)	
**A function is added, thereby halving the spread of infections**							
	No resistance to use	140 (13.4)		118 (11.3)		120 (11.5)	
	Not much resistance to use	304 (29.1)		259 (24.9)		256 (24.7)	
	Some resistance to use	339 (32.5)		369 (35.5)		354 (34.1)	
	Significant resistance to use	152 (14.6)		168 (16.1)		180 (17.3)	
	Absolutely opposed to use	108 (10.4)		127 (12.2)		129 (12.4)	
**Feature additions mean reduced charges for device use**							
	Would accept at a discount of about ¥200 (US $1.48) a month	112 (10.7)		83 (8.0)		88 (8.5)	
	Would accept at a discount of about ¥500 (US $3.70) per month	147 (14.0)		112 (10.7)		97 (9.3)	
	Would accept at a discount of about ¥1000 (US $7.29) per month	154 (14.7)		150 (14.4)		147 (14.1)	
	Would accept at a discount of about ¥2000 (US $14.78) per month	87 (8.3)		123 (11.8)		110 (10.6)	
	Would accept at a discount of about ¥3000 (US $22.17) per month	52 (5)		53 (5.1)		65 (6.3)	
	Would accept at a discount of about ¥5000 (US $36.95) per month	133 (12.7)		138 (13.2)		145 (13.9)	
	I would not want to use it regardless of the discount	362 (34.6)		384 (36.8)		388 (337.3)	

### Factors Associated With the Dependent Variables

The results of the logistic regression of the relationship between those who installed COCOA and the basic characteristics of the respondents are shown in Table 3. There was no significant difference in terms of gender or age groups; regarding the frequency of SNS use, those who used Facebook at least several times per week were more likely to have COCOA installed (odds ratio [OR] 1.5, 95% CI 1.0-2.2). Although there was no significant difference in terms of their own or family members' history of COVID-19 infection, those who answered *strongly agree* or *agree* to the question of whether they feared they might infect others if they contracted COVID-19 were more likely to have installed COCOA (OR 2.2, 95% CI 1.1-4.7). In addition, those who reported adhering to government-mandated behavioral guidelines for infection control were more likely to have installed the app (OR 2.0, 95% CI 1.0-4.0).

The association between the response *absolutely opposed to use* of the contact-confirming app with added functions A, B, and C and the independent variables of the respondents are provided in Table 4. The respondents’ basic characteristics, frequency of SNS use, history of COVID-19 infection, fear of the consequences of being infected themselves, and the degree of adherence to infection control behaviors were not significantly associated with the dependent variables for any of the functions A, B, and C. However, when all functions were added, those who were concerned about leaks or misuse of their personal information were more likely to respond that they were *absolutely opposed to use* of a contact app with more functions added than those who were not concerned (function A: OR 2.1, 95% CI 1.4-3.3; function B: OR 2.3, 95% CI 1.5-3.5; function C: OR 2.5, 95% CI 1.7-3.7). For function B, there was a negative correlation between the answers of *strongly agree* and *agree* in response to the question of whether the national and prefectural governments were controlling the spread of infections with a strong sense of responsibility and the response of *absolutely opposed to use* with the new functions (OR 0.5, 95% CI 0.2-0.9).

**Table 3:** Factors related to COVID-19 Contact-Confirming Application installation.

Independent variables			Odds ratio (95% CI)
Female (reference: male)			0.7 (0.5-1.0)
**Age (years) group (reference: aged <34 years)**			
	35-64	1.0(0.6-1.8)	
	≥65	0.8 (0.4-1.6)	
Living alone (reference: living with others)			1.1 (0.7-1.6)
**SNS^a^ accessed occasionally per week (reference: accessed below this amount)**			
	Twitter	1.1 (0.8-1.6)	
	Facebook	1.5 (1.0-2.2)	
	Instagram	1.3 (0.9-1.9)	
**COVID-19 infection history (reference: no history)**			
	Respondent has a history of COVID-19	1.3 (0.6-2.6)	
	Respondent’s family has a history of COVID-19	1.2 (0.9-1.6)	
**Respondent fears COVID-19 infection (reference: disagree)**			
	Fear of health deterioration to oneself	1.0 (0.6-1.7)	
	Fear of transmission to one's family and friends	0.6 (0.3-1.3)	
	Fear of transmission to people other than family members or friends	2.2 (1.1-4.7)	
**Compliance with preventative behaviors (reference: I do not do this)**			
	Wearing masks when going out	0.7 (0.3-1.6)	
	Restrictions on activities such as meetings	2.0 (1.0-4.0)	
**Assessment of authorities (reference: disagree)**			
	Infection is being controlled through infection countermeasures conducted by authorities	1.3 (0.8-2.0)	
	Authorities are conducting infection control with a sense of responsibility	0.9 (0.6-1.5)	
	I am concerned that my personal information collected by authorities might be leaked or misused for other purposes	0.9 (0.7-1.2)	

^a^SNS: social networking site.

**Table 4:** Factors associated with the response “I am absolutely opposed to using the app” if functions A to C are added.

Independent variables			Function A, odds ratio (95% CI)		Function B, odds ratio (95% CI)		Function C, odds ratio (95% CI)
Female (reference: male)			0.7 (0.5-1.2)		0.8 (0.5-1.2)		0.7 (0.5-1.1)
**Age (years) group (reference: aged <34 years)**							
	35-64	0.9 (0.4-1.8)		1.0 (0.5-2.0)		1.0 (0.5-2.0)	
	≥65	0.7 (0.3-1.7)		0.8 (0.3-1.9)		1.0 (0.4-2.2)	
Living alone (reference: living with others)			0.9 (0.5-1.6)		0.8 (0.5-1.4)		0.9 (0.5-1.5)
**SNS^a^ accessed occasionally per week (reference: accessed below this amount)**							
	Twitter	1.0 (0.6-1.6)		1.0 (0.6-1.7)		1.0 (0.6-1.6)	
	Facebook	0.8 (0.5-1.5)		0.7 (0.4-1.2)		0.7 (0.4-1.2)	
	Instagram	1.2 (0.7-2.2)		1.3 (0.7-2.2)		1.1 (0.7-1.9)	
**COVID-19 infection history (reference: no history)**							
	Respondent has a history of COVID-19	1.5 (0.7-3.4)		1.5 (0.7-3.4)		1.2 (0.5-3.0)	
	Respondent’s family has a history of COVID-19	0.9 (0.6-1.6)		0.9 (0.5-1.5)		0.8 (0.5-1.4)	
**Respondent fears COVID-19 infection (reference: disagree)**							
	Fear of health deterioration to oneself	0.9 (0.5-1.8)		0.9 (0.5-1.7)		0.8 (0.4-1.6)	
	Fear of transmission to one’s family and friends	0.7 (0.3-1.7)		0.8 (0.3-2.0)		0.7 (0.3-1.6)	
	Fear of transmission to people other than family members or friends	0.9 (0.4-2.2)		0.7 (0.3-1.6)		1.0 (0.4-2.3)	
**Compliance with preventative behaviors (reference: I do not do this)**							
	Wearing masks when going out	0.8 (0.3-1.8)		0.8 (0.3-1.8)		0.9 (0.4-2.0)	
	Restrictions on activities such as meetings	0.5 (0.3-1.0)		0.5 (0.3-1.0)		0.5 (0.3-1.0)	
**Assessment of authorities (reference: disagree)**							
	Infection is being controlled through countermeasures conducted by authorities	1.2 (0.6-2.4)		1.3 (0.6-2.6)		1.0 (0.5-2.0)	
	Authorities are controlling the infection with a sense of responsibility	0.6 (0.3-1.1)		0.5 (0.2-0.9)		0.6 (0.3-1.2)	
	I am concerned that my personal information collected by the authorities might be leaked or misused for other purposes	2.1 (1.4-3.3)		2.3 (1.5-3.5)		2.5 (1.7-3.7)	

^a^SNS: social networking site.

We also identified the factors associated with the response *absolutely opposed to use*, even when users were presented with trade-offs between privacy on the one hand and public health benefits from each feature’s addition and the personal benefits of the feature additions in the form of discounted mobile phone use charges on the other hand (Tables 5 and 6, respectively). Even if the addition of each function reduced the spread of infection by half, the factors that were significantly associated with the response *absolutely opposed to use* of the app were the same for all functions (A, B, and C). One of the factors was positively correlated, and two were negatively correlated. The factor that was positively correlated was the respondents who were concerned about data leaks and misuse of personal information (function A: OR 2.2, 95% CI 1.4-3.5; function B: OR 2.2, 95% CI 1.4-3.3; function C: OR 2.5, 95% CI 1.6-3.8). Conversely, the factors that presented negative correlations were adherence to the government-imposed restrictions on activities (function A: OR 0.4, 95% CI 0.2-0.8; function B: OR 0.4, 95% CI 0.2-0.7; function C: OR 0.4, 95% CI 0.2-0.8) and agreement that the national and local governments were conducting infection control with a sense of responsibility (function A: OR 0.4, 95% CI 0.2-0.8; function B: OR 0.5, 95% CI 0.2-1.0; function C: OR 0.5, 95% CI 0.2-0.9).

The only factor that was positively associated with the response *absolutely opposed to use*, even if there was a discount on mobile phone use charges, was concern about leaks and misuse of the collected personal information, which was true for all functions (function A: OR 1.8, 95% CI 1.3-2.4; function B: OR 1.9, 95% CI 1.5-2.6; function C: OR 1.8, 95% CI 1.4-2.4). In contrast, adhering to government restrictions on activities had a negative correlation with the dependent variables for all functional additions (function A: OR 0.5, 95% CI 0.3-0.8; function B: OR 0.5, 95% CI 0.3-0.9; function C OR 0.6, 95% CI 0.3-0.9). Fear of transmission to family members if respondents became infected was negatively correlated with functions A and C (function A: OR 0.5, 95% CI 0.2-0.9; function C: OR 0.5, 95% CI 0.3-0.9).

**Table 5:** Factors associated with the response “I am absolutely opposed to the use of the app” if it is assumed that the spread of infections will be halved by the addition of functions A to C.

Independent variables		Function A, odds ratio (95% CI)	Function B, odds ratio (95% CI)	Function C, odds ratio (95% CI)
Female (reference: male)		0.6 (0.4-1.0)	0.7 (0.5-1.1)	0.7 (0.4-1.0)
**Age (years) group (reference: aged <34 years)**				
	35-64	1.0 (0.4-2.1)	0.8 (0.4-1.5)	0.8 (0.4-1.5)
	≥65	1.0 (0.4-2.6)	0.7 (0.3-1.6)	0.8 (0.4-1.9)
	Living alone (reference: living with others)	1.0 (0.6-1.8)	0.9 (0.5-1.6)	1.0 (0.6-1.7)
**SNS^a^ accessed occasionally per week (reference: accessed below this amount)**				
	Twitter	0.9 (0.5-1.6)	0.8 (0.5-1.4)	0.9 (0.5-1.5)
	Facebook	1.0 (0.5-1.7)	0.8 (0.5-1.5)	0.8 (0.5-1.4)
	Instagram	1.4 (0.8-2.6)	1.5 (0.8-2.6)	1.5 (0.9-2.7)
**COVID-19 infection history (reference: no history)**				
	Respondent has a history of COVID-19	1.6 (0.6-4.4)	1.5 (0.6-4.1)	1.6 (0.7-3.6)
	Respondent’s family has a history of COVID-19	0.6 (0.2-1.4)	0.6 (0.2-1.3)	0.9 (0.5-1.5)
**Respondent fears COVID-19 infection (reference: disagree)**				
	Fear of health deterioration to oneself	0.8 (0.4-1.7)	1.0 (0.5-1.9)	1.0 (0.5-1.9)
	Fear of transmission to one’s family and friends	0.4 (0.1-1.1)	0.5 (0.2-1.3)	0.5 (0.2-1.3)
	Fear of transmission to people other than family members or friends	1.3 (0.5-3.8)	1.0 (0.4-2.5)	1.0 (0.4-2.6)
**Compliance with preventative behaviors (reference: I do not do this)**				
	Wearing masks when going out	1.1 (0.5-2.7)	1.2 (0.5-2.8)	1.0 (0.4-2.4)
	Restrictions on activities such as meetings	0.4 (0.2-0.8)	0.4 (0.2-0.7)	0.4 (0.2-0.8)
**Assessment of authorities (reference: disagree)**				
	Infection is being controlled through infection countermeasures conducted by authorities	1.6 (0.7-3.5)	1.2 (0.6-2.5)	1.2 (0.6-2.5)
	Authorities control infections with a sense of responsibility	0.4 (0.2-0.8)	0.5 (0.2-1.0)	0.5 (0.2-0.9)
	I am concerned that my personal information collected by authorities might be leaked or misused for other purposes	2.2 (1.4-3.5)	2.2 (1.4-3.3)	2.5 (1.6-3.8)

^a^SNS: social networking site.

**Table 6:** Factors associated with the response “I would not want to use the app” when a fee reduction is presented as a trade-off for additional functions A to C.

Independent variables			Function A, odds ratio (95% CI)		Function B, odds ratio (95% CI)		Function C, odds ratio (95% CI)
Female (reference: male)			1.2 (1.0-1.7)		1.2 (0.9-1.6)		1.2 (0.9-1.6)
**Age (years) group (reference: aged <34 years)**							
	35-64 years	0.9 (0.5-1.4)		0.8 (0.5-1.3)		0.9 (0.5-1.4)	
	≥65 years	0.7 (0.4-1.4)		0.7 (0.4-1.2)		0.8 (0.4-1.5)	
Living alone (reference: living with others)			1.1 (0.7-1.5)		1.1 (0.8-1.6)		1.1 (0.7-1.6)
**SNS^a^ accessed occasionally per week (reference: accessed below this amount)**							
	Twitter	0.9 (0.6-1.2)		0.9 (0.6-1.3)		1.0 (0.7-1.4)	
	Facebook	0.9 (0.6-1.4)		0.9 (0.6-1.3)		0.8 (0.5-1.1)	
	Instagram	0.7 (0.4-1.0)		0.8 (0.5-1.1)		0.8 (0.5-1.1)	
**COVID-19 infection history (reference: no history)**							
	Respondent has a history of COVID-19	0.9 (0.4-1.9)		0.8 (0.4-1.8)		0.9 (0.4-2.0)	
	Respondent's family has a history of COVID-19	0.9 (0.6-1.3)		0.9 (0.6-1.3)		0.9 (0.6-1.2)	
**Respondent fears COVID-19 infection (reference: disagree)**							
	Fear of health deterioration to oneself	1.1 (0.7-1.8)		1.0 (0.6-1.6)		1.1 (0.7-1.8)	
	Fear of transmission to one's family and friends	0.5 (0.2-0.9)		0.7 (0.3-1.2)		0.5 (0.3-0.9)	
	Fear of transmission to people other than family members or friends	0.9 (0.5-1.7)		0.8 (0.4-1.4)		1.0 (0.5-1.7)	
**Compliance with preventative behaviors (reference: I do not do this)**							
	Wearing masks when going out	0.9 (0.5-1.9)		0.9 (0.5-1.8)		1.0 (0.5-1.9)	
	Restrictions on activities such as meetings	0.5 (0.3-0.8)		0.5 (0.3-0.9)		0.6 (0.3-0.9)	
**Assessment of authorities (reference: disagree)**							
	Infection is being controlled through infection countermeasures conducted by authorities	0.9 (0.6-1.5)		0.9 (0.6-1.4)		0.8 (0.5-1.3)	
	Authorities control infections with a sense of responsibility	0.7 (0.5-1.1)		0.7 (0.5-1.1)		0.7 (0.5-1.1)	
	I am concerned that my personal information collected by authorities might be leaked or misused for other purposes	1.8 (1.3-2.4)		1.9 (1.5-2.6)		1.8 (1.4-2.4)	

^a^SNS: social networking site.

## Discussion

### Principal Findings

In formulating the hypotheses for this study, we were interested in the public’s attitude toward privacy risks as an inadvertent side effect of the convenience offered by information technology. As Yuan et al [22] suggested, privacy risk strategies are socially implemented after taking into account 2 types of trade-offs that affect individuals’ disclosure behavior: those between the expected benefits of privacy disclosure and the effectiveness of risk-management methods. The contact-tracing app or contact notification app that was developed and used in many countries to prevent the spread of the COVID-19 pandemic epitomizes this theory. The contact notification app (COCOA) in operation in Japan was developed to minimize privacy risks to the public; however, its effectiveness in preventing infection was lower than expected. Therefore, the addition of functions that cannot be performed by the current COCOA, such as the collection of mobile logs and secondary use of the collected data, raises concerns about the increased risk to privacy and the functions themselves. The core objective of our research was to identify how the general public perceives the trade-offs between additional personal and public interest. We also wanted to investigate the extent to which people trade personal privacy risks for economic incentives, as noted by Hann et al [23]. This study was conducted on the assumption that when the administrative body of a country plans to develop and disseminate a contact-confirming app that is more effective in preventing the spread of infectious diseases, citizens who use the app may have some resistance to the government’s collection of activity logs and provision of individual interventions for people with confirmed infections. We also hypothesized that this resistance might be mitigated by emphasizing the public health benefits of an effective contact-confirming app or through a discount on mobile phone use charges for individuals using the app [24,25].

The results of the logistic regression of factors associated with COCOA installation showed a positive correlation with fear of transmission of infection to others in the event of oneself becoming infected; a reasonable interpretation of this result is possible. However, the positive correlation was not significant; rather, the proportion of citizens living in Japan who feel obligated to install COCOA, or who have a high assessment of the app’s usefulness, is considered low.

The survey then introduced functions A, B, and C, which are expected to increase the risk of personal privacy violations concerning the current COCOA, and asked about the resistance to using the app when each of these functions was added. Approximately one-third of all respondents for functions A, B, and C indicated that they felt uncomfortable using the app. This result was more generous than expected at the beginning of the survey. Our initial assumption was that more than half of the survey respondents would react with at least moderate resistance to each of the additional functions. This result would suggest that the public is likely to be more receptive to the use of privacy-related information by the authorities for maintaining public health than we had expected.

Even when function B (ie, location tracking and specific encouragement of activities) were added to function A, there was no significant difference, suggesting that the additional fear and resistance to individual interventions are not necessarily significant. Similarly, there was no significant difference for function C, indicating that the resistance to the collection of personal information for infection prevention measures was not particularly high.

The percentage of respondents who answered *absolutely opposed to use* or *significant resistance to use* of the app decreased slightly when the addition of functions A, B, and C was known to reduce the degree of spread of infectious disease. This finding suggests that increased public health benefits can be traded against personal privacy risks. This result may be more characteristic of the trade-offs of interests in the somewhat special domain of health rather than the general market society principle [26,27]. Surprisingly, when presented with the condition that the addition of a feature would result in a discount on the cell phone bill, one-third of respondents stated that “I would not want to use it regardless of the discount” for all functions A, B, and C. Although it is difficult to explain this result via a literature review, we argue that this result was influenced by the health-related agenda and the fact that the project was a national government agency. When personal privacy is given up, the motivation behind it may not be to make money but to work together to help prevent the spread of infectious diseases. If so, we believe that bringing in financial incentives when promoting such behavior might inadvertently upset citizens' sentiments.

We also deem our interpretation of the results presented in Tables 4-6 to be relevant. Resistance to using apps was not associated with respondents’ basic characteristics, an affinity for SNS, or history of COVID-19 but was negatively correlated with their evaluation of the executive branch regarding infection prevention activities and compliance concerning infection prevention behaviors. We interpreted this result as suggesting that the behavior of using exposure notification apps has an element of altruistic behavior that seeks the public good [28,29]. In contrast, as mentioned many times in previous literature, the sense of risk regarding information leakage had the highest contribution rate among the factors related to resistance to app use [16-18,30-32].

### Generalizability

It may be acceptable for society to allow public organizations, such as national government agencies, to collect and use individuals’ private information for specific purposes as part of nationwide efforts to control infectious diseases. However, such interventions must be balanced by the resistance felt by the public regarding the provision of private information [33]. In addition, it is necessary to have a continuous discussion between administrative agencies and the public regarding the conditions that would enable the formation of a social consensus on the collection and use of private information for administrative purposes. In this study, we presented a hypothetical basis for this discussion. The current COCOA in Japan is one of the weakest interventions for the collection and use of private information; however, the public response is that certain prerequisites must be in place, such as a clear purpose of use and a robust security environment. If these factors are satisfactorily explained to the public, the intervention will be stronger; however, even if this occurs, the possibility that the intervention will be accepted is still low [34]. For example, it may be possible to actively consider the collection of activity logs using the GPS function on people’s phones [35,36]. However, these activities are tolerated by the public as they are aware of the greater public health risks posed by a global crisis such as the COVID-19 pandemic; a more cautious attitude is necessary when considering other general administration purposes [37,38].

An important interpretation to note is that there is no trade-off between the permissibility of government interventions and the provision of special incentives to individuals, such as discounts on mobile phone bills. This study revealed that such simplistic benefits may upset public sentiments and increase suspicion of the government. In a future society where personal health records are widely distributed, public organizations will increasingly require access to private information on the cloud. In such a case, it is more important for public organizations to increase the general benefits of managing personal information and provide assurance that this is being done safely rather than providing incentives to individuals who provide such personal information.

### Study Limitations

This survey involved a representative sample of the Japanese population. The response rate was >10/13. The results could potentially be a decision resource for the government when considering whether to add more functions to the app.

However, 4 limitations must be noted. First, it is possible that the public did not understand the functions of the current COCOA. The questions asked in the survey assumed that additional functions would be added to the current COCOA, which may have been difficult for respondents to envision, along with the advantages and disadvantages that would result for themselves and the public. Second, as this was a quantitative survey based on a specific hypothesis, it was not possible to set out the specific functions that the public would expect from COCOA. A survey with a qualitative approach would be necessary to explore the functions. Third, we were unable to examine the psychometric properties of the attitudes measured in this study. Usually, when attitudes are measured using questionnaires, their reliability and validity must be ensured. However, in this study, the lack of existing general-purpose scales and insufficient time for scale development created major limitations in interpreting the results. The existing literature identifies the main element of the public’s resistance to the use of contact-tracing apps as a feeling of insecurity regarding the leakage of information or its use for purposes other than as intended [39,40]. As such an attitude is a very important concept for the future *Ethical, Legal, and Social Issues in Science and Technology* agenda in information technology, it may be necessary to develop a common rating scale with guaranteed psychometric validity [41,42]. Fourth, when interpreting the results of the survey, the impact of the *privacy paradox* along with clinical relevance must be considered. The privacy paradox is a situation in which people express concerns about privacy through their attitudes but do not hesitate to take actions that pose a significant risk in terms of revealing their private information in real life [43,44]. To avoid this paradox, we asked about respondents’ attitudes regarding their actual behaviors rather than their conceptual concerns. However, when reflecting on the results of this study for policy recommendations, it must be stated that the evidence is influenced by this paradox.

### Conclusions

In this study, we investigated the public’s attitude toward the addition of several functions to COCOA, an app used to confirm COVID-19 contact distributed by the Japanese government. Although there was some resistance to its official and governmental release and use, there was little change in the public’s resistance, as depicted in this study, regardless of the added functions’ content. However, the sense of resistance to the government’s intervention was not ameliorated by the incentives provided to individuals. On the basis of the results of this study, we believe that it is acceptable for citizens to implement functions that involve a certain degree of privacy risk when digitally processed personal privacy information is used primarily to promote health benefits, such as preventing the spread of infectious diseases, provided that the entities in charge of such projects, such as government agencies, are fully accountable for their actions. Furthermore, at least with respect to projects such as health policy, citizens will set aside their privacy concerns in view of broader public health interests rather than individual economic incentives. Thus, this study offers insights into potential strategies for governmental use of private information through mobile devices. Further social research and empirical evidence are needed on this topic to form an *ecosystem* of information and people, which is still expanding daily.

## Appendix

Multimedia Appendix 1 Specific questions on the survey.

## Abbreviations

COCOA: COVID-19 Contact-Confirming Application
OR: odds ratio
SNS: social networking site
