# Supplementary material for: Public Attitudes Regarding Trade-offs Between the Functional Aspects of a Contact-Confirming App for COVID-19 Infection Control and the Benefits to Individuals and Public Health: Cross-sectional Survey
Source: JMIR Form Res. 2022 Jul 20;6(7):e37720. doi: 10.2196/37720 (PMC9302613; doi:10.2196/37720)
Supplement: Multimedia Appendix 1 [file formative_v6i7e37720_app1.docx]

## Appendix. List of Questions

### Basic characteristics

▪Gender ▪ Age group ▪ Occupation ▪ Current marital status ▪ Living with a person who requires care

Frequency of use of information services

▪Twitter　　▪Facebook　　▪Instagram

### Questions related to COVID-19 infection

Have you ever been diagnosed with COVID-19？

▪Yourself

▪Family or close friends

In the midst of the COVID-19 epidemic, how fearful are you about the following if you yourself were infected？

▪Harm to own health

▪Unknowingly infecting family and friends

▪Unknowingly infecting others (apart from family and friends)

How strictly do you adhere to the following guidelines issued by the government?

▪Wearing masks when going out

▪Restrictions on activities such as outings, dinners, meetings, etc.

What are your thoughts on the infection prevention strategies implemented by the Ministry of Health, Labor and Welfare (MHLW) and other national and local government agencies, as well as on the provision of medical care in hospitals and other facilities?

The spread of novel coronavirus infections has been controlled by measures taken by the following government agencies.

▪County and prefectures

▪Municipal regional health centers

▪Medical facilities such as hospitals and neighborhood clinics

The following government agencies have a strong sense of responsibility for the control of novel coronavirus infections.

▪County and prefectures

▪Municipal regional health centers

▪Medical facilities such as hospitals and neighborhood clinics

There is concern that personal information and other data collected as part of measures against new coronavirus infection by national and prefectural administrative agencies may be leaked or misused for other purposes.

### Questions about the Contact Confirmation Application for Novel Coronavirus (COCOA) provided by the Ministry of Health, Labor and Welfare

Features currently implemented in COCOA

◆If you have tested positive for COVID-19 infection, a positive registration will be made with your consent.

◆If you have been in contact with a positive person at a short distance and for a certain period of time, you will be notified of the contact through the app. Notification of contact only mentions the "date and time of contact," and location details are not received.

◆Those who have been notified of contact will be provided with subsequent contact and consultation information through the application.

◆Neither the positive result nor the contact will be known to the administrator (Ministry of Health, Labor and Welfare) in terms of phone numbers, e-mail addresses, etc.

### What is the installation status of COCOA on your mobile device?

In each of the following cases, please describe your resistance to using this application.

As a precondition, even today, information on COVID-19 infections is reported to the Ministry of Health, Labor and Welfare by hospitals and other medical institutions in accordance with laws and regulations.

Function A:　If you visit a medical institution and are found to be infected with the novel coronavirus, the Ministry of Health, Labor and Welfare (MHLW) is informed by the medical institution, and it then automatically registers you as an infected person in the COCOA application, which is also reflected in the contact person notification function.

Function B:　In addition to "Function A," the MHLW will use the location information function of your smartphone to track whether you and those with whom you come into contact are taking necessary actions to prevent infectious diseases (for example, whether your contacts visit a medical institution afterwards) and, if necessary, the MHLW will prompt you through the application to take specific actions such as requesting medical examinations.

Function C: Although there is no special contact with you or anyone you come in contact with, all information on the movements of people who have installed the application (taking a train, going to a restaurant, etc.) will be recorded through the mobile device, and after being consolidated by the MHLW, this information will be collected by the ministry and used as data for planning and implementing specific infection prevention measures.

### When functions A to C are added individually

If half of the population uses the app with the added functions A through C, it will greatly reduce the spread of infectious diseases in our country compared to if they do not use the app (the app will reduce the number of infections from about 1,000 per day without the app to only 500 per day—half the number that would occur in the absence of the app). If you knew that the app could reduce the spread of infectious diseases in our country, how reluctant would you be to use it?

If you were offered a discount on the monthly fee for a mobile device in exchange for the addition of a new feature, how much discount would you accept for using the application?
